# ARD1-mediated aurora kinase A acetylation promotes cell proliferation and migration

**DOI:** 10.18632/oncotarget.19332

**Published:** 2017-07-18

**Authors:** Tam Thuy Lu Vo, Ji-Hyeon Park, Ji Hae Seo, Eun Ji Lee, Hoon Choi, Sung-Jin Bae, Hoang Le, Sunho An, Hye Shin Lee, Hee-Jun Wee, Kyu-Won Kim

**Affiliations:** ^1^ SNU-Harvard NeuroVascular Protection Research Center, College of Pharmacy and The Research Institute of Pharmaceutical Sciences, Seoul National University, Seoul 08826, Korea; ^2^ Department of Biochemistry, School of Medicine, Keimyung University, Daegu 42601, Korea; ^3^ Crop Biotechnology Institute, GreenBio Science and Technology, Seoul National University, Pyeongchang 25354, Korea

**Keywords:** aurora kinase A, ARD1, lysine acetylation, cell proliferation, cell migration

## Abstract

Aurora kinase A (AuA) is a prerequisite for centrosome maturation, separation, and mitotic spindle assembly, thus, it is essential for cell cycle regulation. Overexpression of AuA is implicated in poor prognosis of many types of cancer. However, the regulatory mechanisms underlying the functions of AuA are still not fully understood. Here, we report that AuA colocalizes with arrest defective protein 1 (ARD1) acetyltransferase during cell division and cell migration. Additionally, AuA is acetylated by ARD1 at lysine residues at positions 75 and 125. The double mutations at K75/K125 abolished the kinase activity of AuA. Moreover, the double mutant AuA exhibited diminished ability to promote cell proliferation and cell migration. Mechanistic studies revealed that AuA acetylation at K75/K125 promoted cell proliferation via activation of cyclin E/CDK2 and cyclin B1. In addition, AuA acetylation stimulated cell migration by activating the p38/AKT/MMP-2 pathway. Our findings indicate that ARD1-mediated acetylation of AuA enhances cell proliferation and migration, and probably contributes to cancer development.

## INTRODUCTION

Defective control of cell proliferation is the main characteristic of cancer. In normal cells, cell division is rigorously controlled by complex signaling circuits. However, cancer cells can sustain proliferative signaling themselves and can grow unstoppably. Another hallmark of cancer is cell migration [1]. Cell migration is a highly integrated process, and controlled by dynamic regulatory mechanisms [2]. Hence, understanding the mechanisms of controlling cell proliferation and cell motility could open new horizons in cancer therapy.

Aurora kinase A (AuA) is a member of the serine/threonine kinase family, which has been implicated in controlling the cell cycle and cell division [3, 4]. The AuA-encoding gene is located on chromosome 20q13.2-q13.33, a region frequently amplified in many cancers such as rectal, ovarian, prostate, colon, and breast cancers [5–7]. The catalytic domain of AuA shares 70% homology with that of other members of the Aurora kinase family, whereas the N-terminal regulatory domain is distinct. The regulatory domain of AuA is responsible for its binding with protein partners. AuA kinase activity has been extensively studied for its vital role in centrosome functions as well as mitotic regulation [4, 8, 9]. AuA kinase is activated by phosphorylation at threonine 288 by TPX2 [10–12]. AuA then phosphorylates the cell cycle regulator cyclin B1/CDK1, promoting mitotic entry [13–15]. Because of the overexpression of AuA in many types of metastatic cancers and its correlation with poor prognosis [16–20], the non-mitotic functions of AuA, in addition to its role in mitotic regulation, have been elucidated. Recent findings have revealed the contribution of AuA in cell migration and metastasis enhancement. AuA reportedly activates cell migration by modulating MMP-2 expression [21]. However, the mechanisms regulating AuA activity in cell movement are still unclear.

Acetylation of lysine residues is one of the major essential post-translational modifications [22], and plays a significant role in modulating histone modification, thus regulating a large number of cellular events. In addition, lysine acetylation modification of non-histone proteins has been shown to be related to several regulatory mechanisms in both healthy and pathological cells [23]. The enzymes responsible for this modification are identified as lysine acetyltransferases. ARD1 is identified as a catalytic subunit of *N*-acetyltransferase [24], and acts on various substrates related to cell homeostasis, cell movement, and cancer development, such as HSP70 [25], myosin light chain kinase [26], and β-catenin [27]. Furthermore, ARD1 can acetylate itself and this autoacetylation of ARD1 is crucial for its activation [28]. ARD1, therefore, plays important roles in many biological processes.

In this study, we identified a previously undefined post-translational modification of AuA by ARD1 acetyltransferase. We found that AuA is acetylated at lysines at positions 75 and 125, consequently this contributes to AuA catalytic capacity, then leading to promotion of cell proliferation and stimulation of cell migration.

## RESULTS

### AuA and ARD1 colocalize during cell division and cell migration

**Figure 1:**
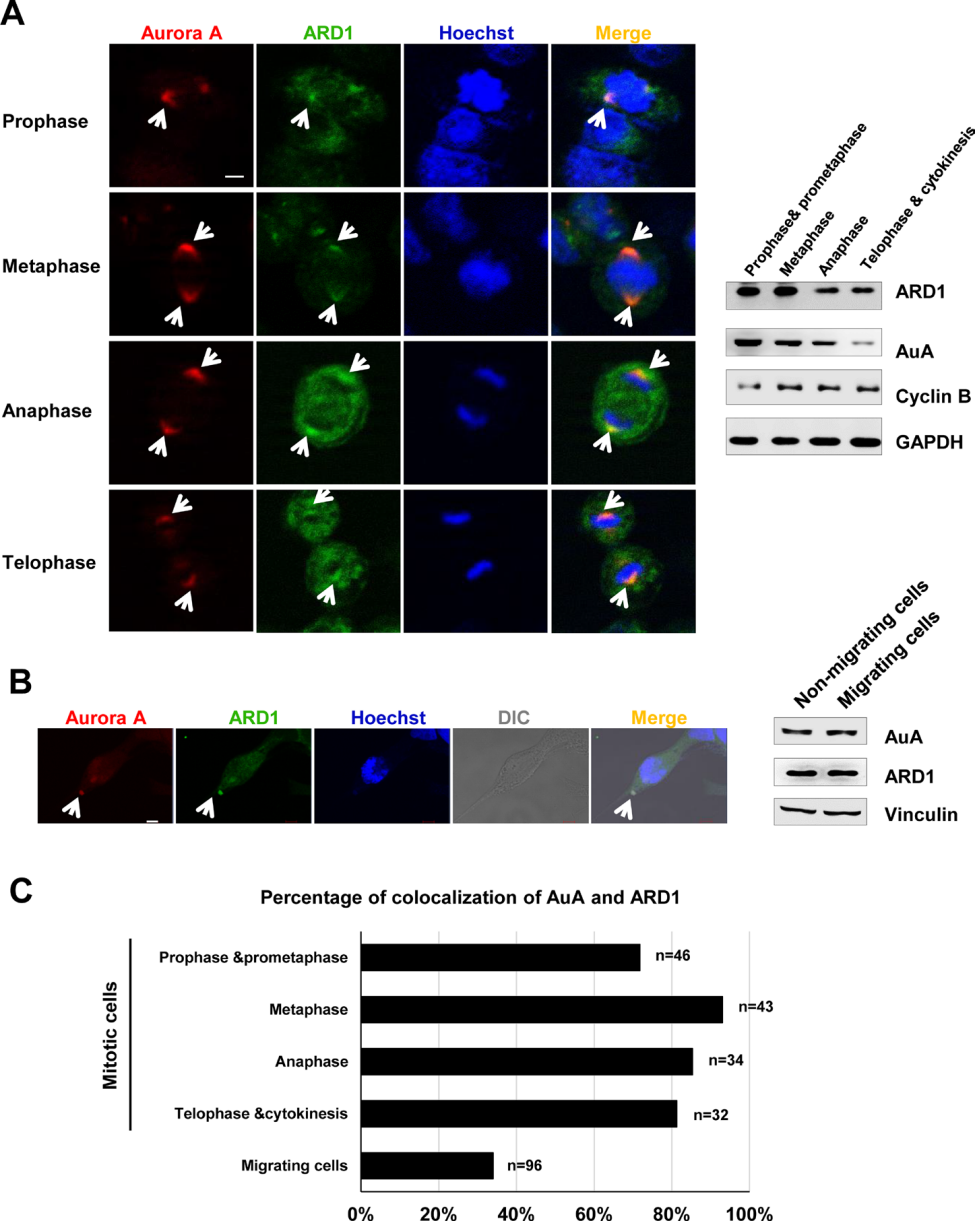
Aurora kinase A colocalalizes with ARD1 during cell division and cell migration (**A**) Left, Aurora A colocalizes with ARD1 at centrosomes during cell division in prophase, metaphase, anaphase and telophase. GFP-ARD1 overexpressing cells (green) were synchronized and stained with anti-AuA antibody (red). DNA was counter-stained by Hoechst. Cells were then viewed under a confocal microscope. Scale bar, 5 μm. Colocalization of ARD1 and AuA is indicated by arrows. Right, Expression of ARD1 and AuA during cell cycle progression. Cyclin B1 was used as the markers of the cell cycle phases. GAPDH was used as loading control. (**B**) Left, Aurora A colocalizes with ARD1 in migrating cell. Monolayer of GFP-ARD1 overexpressing cells (green) on coverslip was scratched to stimulate migration, then cells were stained with anti-AuA antibody (red). DNA was counter-stained by Hoechst. Scale bar, 5 μm. Colocalization of ARD1 and AuA is indicated by arrows. Right, Expression of ARD1 and AuA during cell migration. Vinculin was used as loading control. (**C**) The percentage of cells exhibit the colocalization of ARD1 and AuA during the cell cycle progression and cell migration.

Recent studies showed that ARD1 is essential for cell survival, as knockdown of ARD1 in human cells triggers apoptosis and G1 arrest [27, 29]. We observed the centrosome-like localization of ARD1 (Supplementary Figure 1), and this prompted us to examine whether ARD1 localized to the centrosome. AuA is considered as a centrosome marker owing to its localization to the centrosome and its function in centrosome maturation and separation [30, 31]. Interestingly, ARD1 colocalized with AuA during the cell cycle progression, particularly during the cell division (Figure 1A, 1C). The centrosome not only participates in cell cycle progression, but also contributes to cell migration by undergoing reorientation and supporting the forces generated in cells during cell migration [32]. AuA, as a component of the centrosome, plays a part during cell migration. Here, we also observed the colocalization of ARD1 and AuA in migrating cells (Figure 1B, 1C). These observations suggest that interaction between ARD1 and AuA at the centrosome may play a critical role in cell growth and cell movement.

### Acetylation of AuA is regulated by ARD1

**Figure 2:**
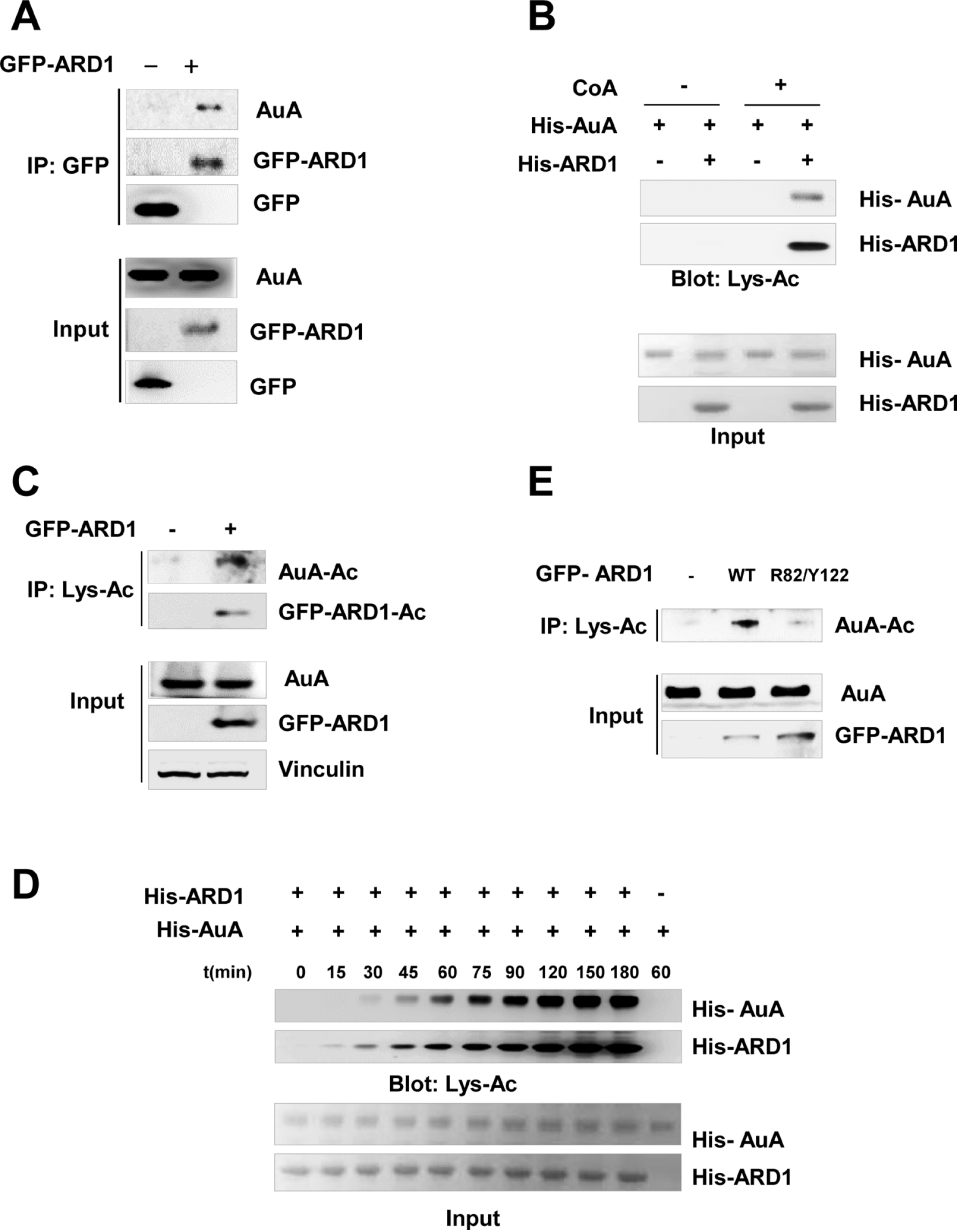
Aurora A is acetylated by ARD1 (**A**) AuA interacts with ARD1. Lysates from HEK293T cells overexpressing GFP-ARD1 were immunoprecipitated with anti-GFP antibody and immunoblotted with anti-AuA antibody or anti-GFP antibody. The experiments were performed at least three times independently. (**B**) AuA is acetylated by ARD1 *in vitro*. His-ARD1, His-AuA recombinants were subjected to *in vitro* acetylation assays with or without presence of acetyl group donor acetyl- coenzyme A (CoA) for 1 h, and acetylation levels of recombinants were assessed by western blotting using an anti-acetylated lysine antibody (Lys-Ac). Ponceau S staining shows the quantification of the input proteins. The experiments were performed at least three times independently. (**C**) Acetylated AuA level increases in GFP-ARD1 overexpressing cells. Lysates from GFP-ARD1 overexpressing MCF7 cells were immuprecipitated with anti-Lys-Ac antibody and analyzed by immunoblotting with anti-AuA antibody or anti-GFP antibody. The experiments were performed at least three times independently. (**D**) AuA acetylation occurs in a time-dependent manner. His-ARD1 recombinants were subjected to *in vitro* acetylation assays for series of time, and acetylation levels of recombinants were assessed by western blotting using an anti-Lys-Ac antibody. Quantification of the input proteins were analyzed by Ponceau S staining. The experiments were performed at least three times independently. (**E**) AuA acetylation is dependent on ARD1 acetyltransferase activity. MCF7 cells were transfected with wild type (WT) GFP-ARD1 or GFP-ARD1 R82F/Y122A mutant. The extracts from the overexpressing cells were immoprecipitated with anti Lys-Ac antibody and acetylated AuA levels were analyzed by immunoblotting with anti-AuA antibody. The experiments were performed at least three times independently.

To determine whether AuA and ARD1 physically interact in cells, we immunoprecipitated ARD1 from lysates of cells overexpressing ARD1 and found that ARD1 was bound to AuA (Figure 2A). As ARD1 is an acetyltransferase that catalyzes the transfer of acetyl groups from acetyl-coA onto lysine residues in its substrates, we hypothesized that AuA could be modulated by ARD1 by acetylation. To examine this hypothesis, we performed an *in vitro* acetylation assay in which recombinant His-tagged AuA was mixed with recombinant His-tagged ARD1 in the presence of acetyl-CoA. Expectedly, AuA was acetylated by ARD1 (Figure 2B). Consistent with the *in vitro* experiment, the overexpression of ARD1 significantly upregulated the level of AuA acetylation in cells (Figure 2C). Interestingly, AuA acetylation occurred in a time-dependent manner after autoacetylation of ARD1 (Figure 2D), suggesting that the autoacetylation of ARD1 is essential for regulating AuA acetylation. Previously, we reported that ARD1, in addition to acetylating a variety of substrates, undergoes self-acetylation and that arginine 82 (R82) and tyrosine 122 (Y122) are required for its acetyltransferase activity [28]. Thus, we examined the levels of AuA acetylation in the presence of functional (wild-type) and R82A/Y122F mutant ARD1 proteins. It was seen that the AuA acetylation level decreased dramatically when ARD1 was mutated at R82 and Y122 (Figure 2E). Taken together, these data indicate that AuA interacts with ARD1, and AuA acetylation is regulated by functional ARD1.

### Lysine residues at positions 75 and 125 of AuA are acetylated by ARD1

**Figure 3:**
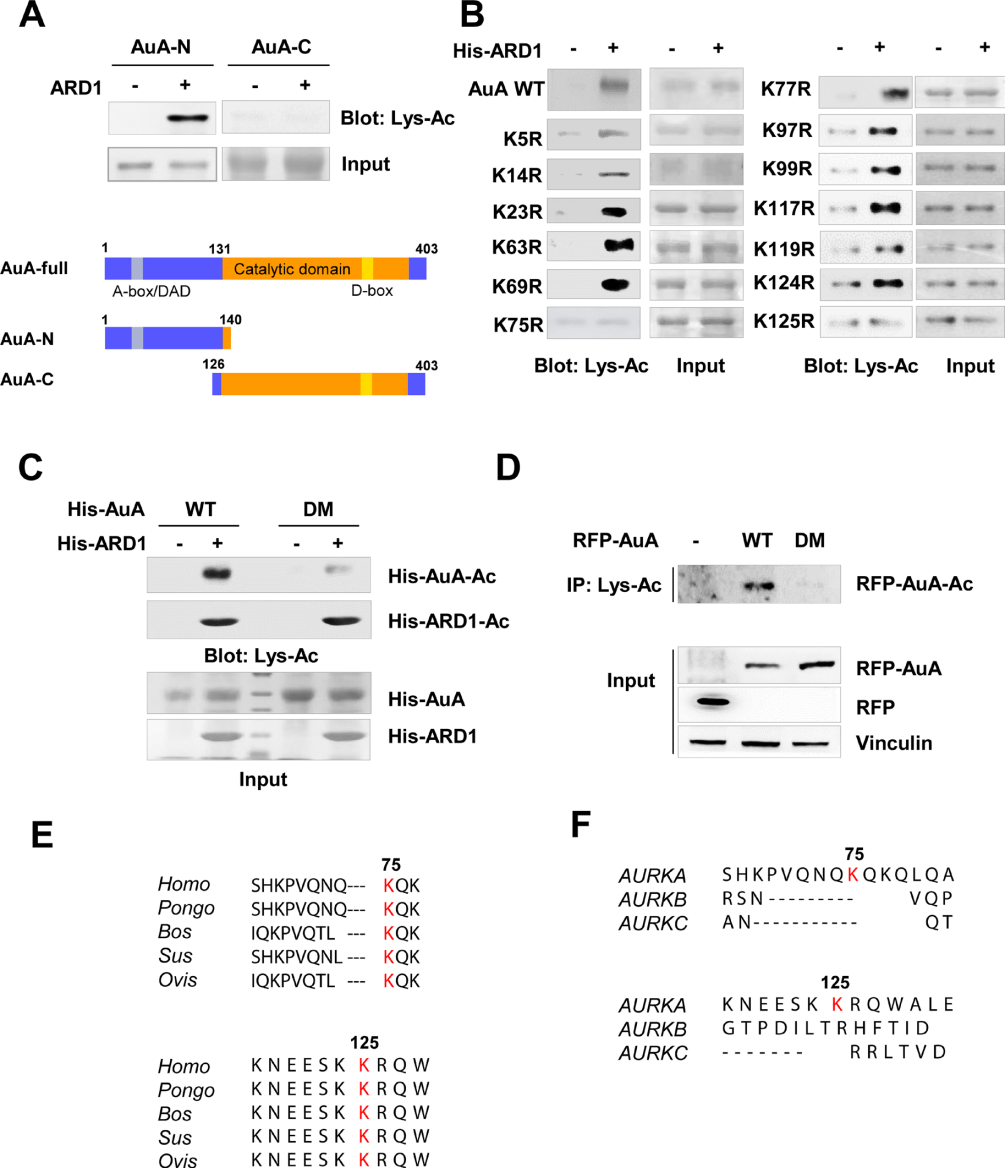
K75, K125 of Aurora A are acetylated by ARD1 (**A**) ARD1 acetylates the N-terminus of AuA *in vitro*. Top, deletion mutants of His-AuA were subjected to *in vitro* acetylation assays with ARD1 recombinant protein. Bottom, construction of AuA deletion mutants. AuA-N, AuA N-terminal domain; AuA-C, AuA C-terminal domain. (**B**) Lysines at positions 75 and 125 of AuA are acetylated by ARD1. Mutagenesis was performed to substitute lysine residues in N-terminus by arginine. Site-mutated AuA recombinants were then applied to *in vitro* assays. The experiments were performed at least three times independently. (**C**) Double mutant AuA K75R/K125R exhibits the negligible acetylation. His-AuA wild type (WT) and K75R/K125R double mutant (DM) were subjected to *in vitro* acetylation assays with His-ARD1. Acetylation levels were assessed by western blot. Each experiment was repeated at least three times. (**D**) Double mutant AuA K75R/K125R displays significantly decreased level of acetylated AuA in cells. The lysates from stable cells overexpressing RFP-AuA WT or RFP-AuA K75R/K125R mutant were collected for immunoprecipitation using anti-Lys-Ac antibody. The immunoprecipitates were examined for acetylated AuA by blotting with anti-AuA antibody. Each experiment was repeated three times independently. (**E**) The lysine residues at positions 75 and 125 are conserved across species. Protein sequence alignment of AuA in various species. (**F**) The lysine residues at positions 75 and 125 are not conserved among Aurora kinase family proteins. Protein sequence alignment of AuA in Aurora kinase family proteins.

AuA comprises 403 amino acids and has two domains, an N-terminal domain spanning residues 1 to 131, and a C-terminal domain spanning residues 132 to 403. The C-terminus includes a catalytic domain that harbors the kinase activity and a destruction box (D-box) that plays a role in ubiquitin-mediated degradation of several mitotic proteins. The N-terminus contains the A-box/D-box activating domain (DAD) that controls AuA degradation (Figure 3A). However, the function of the N-terminal domain is yet unclear [4, 8]. To identify the target sites on AuA that are acetylated by ARD1, we performed *in vitro* acetylation assays with recombinant AuA. For this, we constructed two truncated fragments of AuA, an N-terminal domain-containing fragment comprising amino acids 1 to 140 and a C-terminal domain-containing fragment comprising residues 126 to 403 (Figure 3A). As shown in Figure 3A, the N-terminal domain of AuA was acetylated, but not the C-terminal domain. To further delineate the residues involved in ARD1-mediated AuA acetylation, a series of N-terminal fragments were generated, in which the lysine residues were substituted with arginine to mimic non-acetylated lysine, and *in vitro* acetylation assays were performed. Lysines at positions 75 and 125 were identified as preferable sites for AuA acetylation (Figure 3B). Indeed, AuA acetylation was almost negligible when the K75R/K125R double mutant AuA was subjected to *in vitro* acetylation (Figure 3C). Similarly, cells overexpressing AuA double mutant K75R/K125R displayed a dramatically decreased level of acetylated AuA (Figure 3D), suggesting that these sites are critical for the acetylation of AuA by ARD1. These two sites are conserved across species (Figure 3E), indicating that these sites may be essential for regulating AuA activity. However, the sites are not conserved in other members of the Aurora kinase family (Figure 3F), and are probably responsible for the distinct functions of AuA compared to those of other Aurora kinases in regulating cellular processes.

### ARD1-mediated AuA acetylation at K75/K125 enhances AuA kinase activity

**Figure 4:**
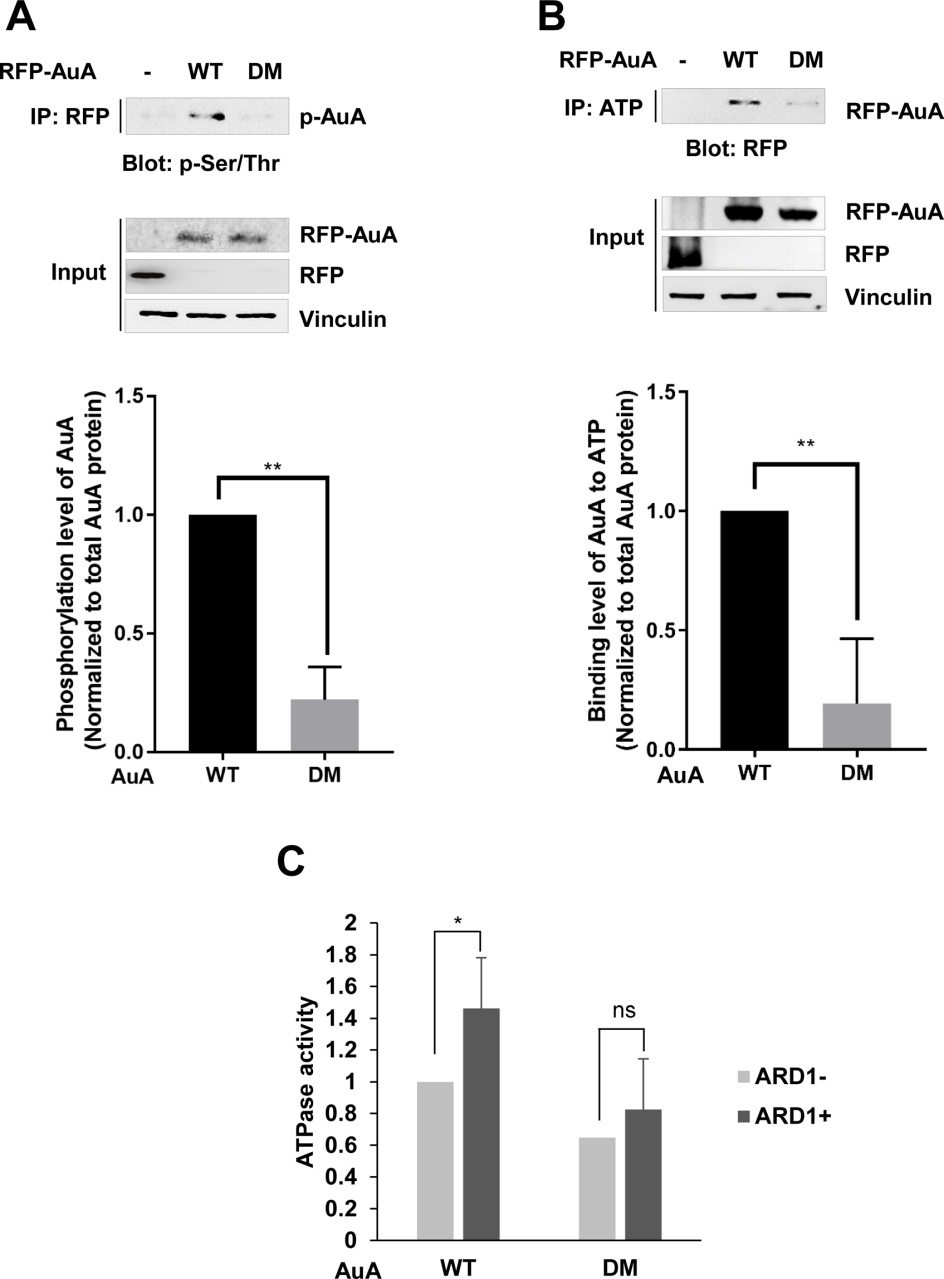
ARD1-mediated AuA acetylation at K75/K125 enhances AuA kinase activity (**A**) Phosphorylation level of AuA is decreased in cells overexpressing K75R/K125R mutant. Top, the collected extracts from MCF7 cells overexpressing RFP-AuA WT or DM were immunoprecipitated with anti-RFP antibody and were then immnublotted with anti-phospho- Serine/Threonine antibody (p-Ser/Thr) for examining phosphorylated AuA. Bottom, phosphorylated AuA levels were analyzed using ImageJ program. **p < 0.005. Error bar indicates S.E.M (*n* = 3). (**B**) ATP-binding ability of AuA mutant is decreased. Lysates of RFP-AuA WT overexpressing cells and RFP-AuA K75R/125R overexpressing cells were incubated with ATP-agarose for 4 h, and ability of AuA binding to ATP was analyzed by western blot using anti-RFP tag antibody. Bottom, ATP-binding capacity was analyzed from western blot density using Image J program. **p < 0.005. Error bar indicates S.E.M (*n* = 3). (**C**) Acetylation of AuA increases ATPase activity. His-AuA WT and His-AuA DM recombinants were acetylated, and subjected to reaction with ATP. ATPase activity was analyzed by measuring absorbance at 595 nm. *p < 0.05; n.s, no significant. Error bar indicates S.E.M (*n* = 3).

The functions of AuA in diverse cellular processes are related to its kinase activity. Several studies have proposed that phosphorylation affects AuA kinase activity. To investigate whether AuA acetylation influences its catalytic activity, we examined the phosphorylation level of AuA. Impressively, AuA phosphorylation was elevated in stable cells overexpressing wild-type (WT) AuA, but not in cells expressing the K75R/K125R mutant AuA (Figure 4A). AuA catalytic activity is strictly governed by an ATP cycle comprising ATP binding, ATP hydrolysis, and release of Pi and ADP [33, 34]. This ATP cycle is regulated by the ATP-binding site of AuA, which is located at the interface of the catalytic core [34]. Thus, the ability of AuA to bind ATP is critical for AuA activation. To elucidate the impact of AuA acetylation on its kinase activity, we next probed the ability of WT and K75R/K125R mutant AuA to bind ATP. We found that K75R/K125R mutant AuA exhibited significantly reduced ATP-binding capacity than the WT protein (Figure 4B). Furthermore, the ATPase activity of WT AuA was significantly enhanced in the presence ARD1, which acetylates AuA; however, the mutant AuA, which was unable to be acetylated, did not show significant change in ATPase activity (Figure 4C), indicating that ATP hydrolysis by acetylated AuA is more efficient than that by the non-acetylated mutant. Taken together, these data suggest that ARD1-mediated AuA acetylation at K75/K125 positively regulates AuA kinase activity.

### ARD1-mediated AuA acetylation enhances cell proliferation

**Figure 5:**
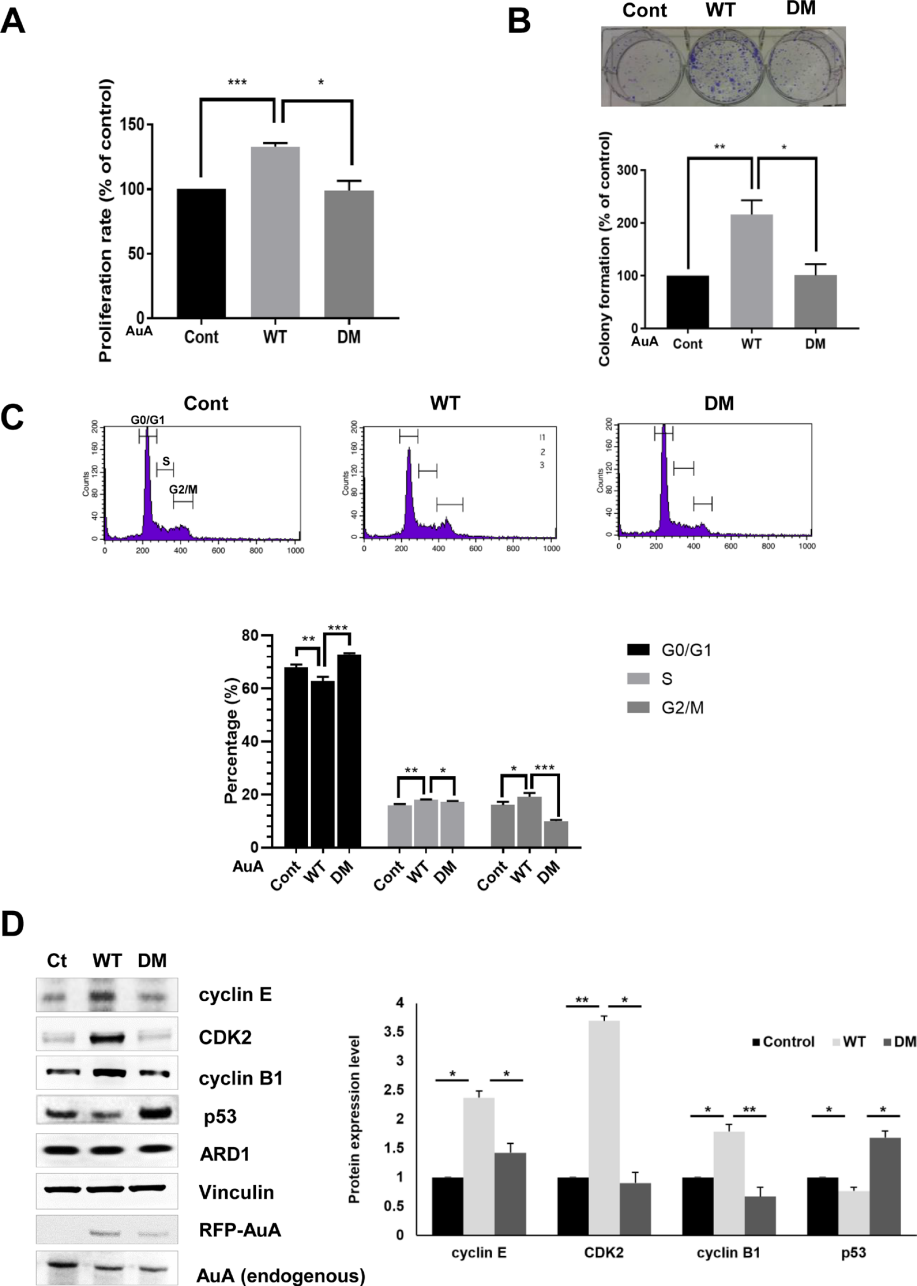
ARD1-mediated AuA acetylation enhances cell proliferation (**A**) Acetylation of AuA increases cell proliferation. The overexpressing RFP-AuA WT and RFP-AuA K75R/K125R MCF7 cells were seeded in 96-well plates and cultured for 48 h. Cells were then treated with MTT and measured absorbance at 490nm. ***p < 0.0005; *p < 0.05. Error bar indicates S.E.M (*n* = 3). (**B**) Acetylation of AuA promotes colony formation of MCF7 cells. The overexpressing RFP-AuA WT and RFP-AuA K75/125 MCF7 cells were seeded in 6-well plates and cultured for 7 days. Cells were then fixed with formaldehyde and stained with crystal violet to visualize colony formation for quantification. Representative image is shown. **p < 0.005, *p < 0.05. Error bar indicates S.E.M (*n* = 3). (**C**) The overexpressing RFP-AuA WT cells shows the decreased population in G0/G1 phase and increased number of cells in S phase and G2/M phase. RFP-AuA WT and RFP-AuA K75R/K125R overexpressing MCF7 cells were stained with propidium iodide and analyzed by BD Calibur flowcytometry. ***p < 0.0005; **p < 0.005; *p < 0.05. Error bar indicates S.E.M (*n* = 3). (**D**) Acetylation of AuA increases expression level of cyclin B1, cyclin E and CDK2, whereas decreases expression level of p53. Expression level of target proteins were analyzed by western blot and quantified by Image J program. Vinculin was used as loading control. *p < 0.05; **p < 0.005. Error bar indicates S.E.M (*n* = 3).

AuA is a key regulator of centrosome maturation and separation, in preparation for cell division. Moreover, various mitotic proteins are regulated by AuA-induced phosphorylation to ensure chromosome segregation and cell cycle progression. Overexpression or downregulation of AuA causes inappropriate cell cycle progression, leading to promotion or inhibition of cell growth. Because acetylation of AuA enhanced AuA kinase activity, we sought to elucidate the role of AuA acetylation in cell proliferation. We seeded MCF7 cells in 96-well plates, incubated the cells for 48 h, treated them with MTT, and measured absorbance as an indicator of cell density. After 48 h, cells overexpressing WT AuA showed significant proliferation compared with cells overexpressing K75R/K125R mutant AuA (Figure 5A). In confirmation with this data, clonogenic assays displayed the outgrowth of cells expressing acetylated AuA compared with cells expressing nonacetylated mutant AuA (Figure 5B). Then we checked distribution of cells in phases of cell cycle by the flowcytometry. Population of G0/G1 phase of AuA WT overexpressing cells decreased, whereas the number of cells in G2/M phase significantly increased, accompanying with the slight increase in S phase, indicating that acetylated AuA overexpressing cells had higher proliferative proportion than that of non-acetylated AuA mutant overexpressing cells (Figure 5C). Normal cell proliferation requires a balance between cell division and cell death. Dysregulation of either or both of these processes causes abnormal cell proliferation in various diseases. Cell division is rigorously controlled by checkpoints at cell cycle stage transitions. Because of the important aspect of AuA in cell cycle regulation, we asked whether acetylation modification of AuA could be related to cell cycle regulation via cyclins. We found that cyclin E/CDK2 and cyclin B, which are regulators of G1/S and G2/M checkpoints, respectively, were dramatically upregulated in WT AuA-overexpressing cells, whereas the cells that overexpressed non-acetylated mimic mutation of AuA did not increase the expression levels of cyclin E/CDK2 and cyclin B1, suggesting that the acetylation of AuA at K75/K125 upregulate cyclin E/CDK2 and cyclin B1 expression (Figure 5D). Meanwhile, the expression level of p53 was downregulated in cells overexpressing WT AuA, but elevated in cells overexpressing mutant AuA. This result is consistent with that of a previous study, which showed that AuA-induced p53 phosphorylation triggers p53 degradation [35]. Collectively, these results reveal that AuA acetylation at K75/K125 enhances cell proliferation via cell cycle acceleration.

### K75/K125 acetylation of AuA promotes cell migration

**Figure 6:**
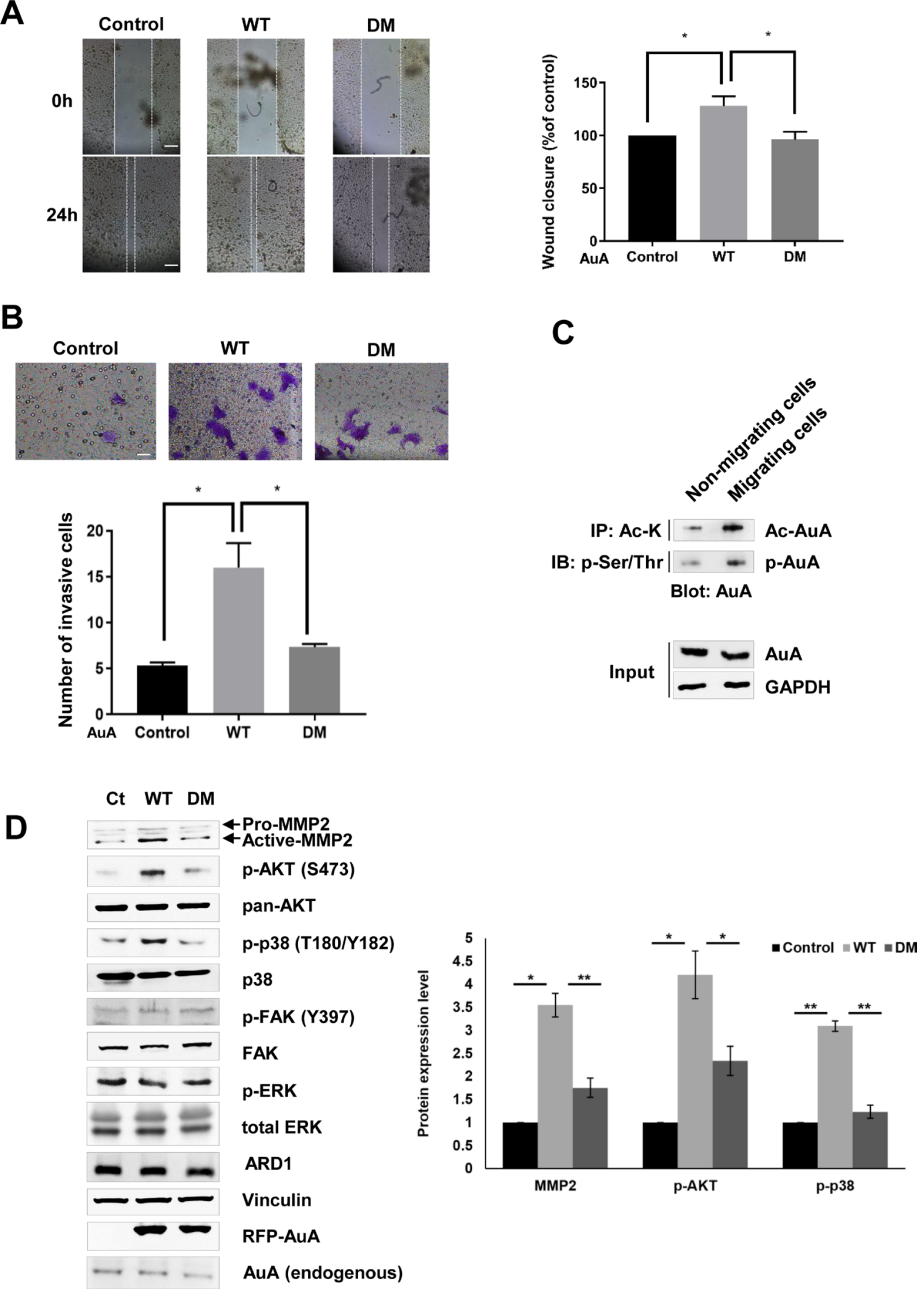
K75/K125 acetylation of AuA promotes cell migration (**A**) Acetylation of AuA promotes cell migration. Wound healing assay of MCF7 cells was performed, the closure ratios were analyzed after 24 h.*p < 0.05. Error bar indicates S.E.M (*n* = 3). Scale bar, 100 μm. (**B**) Acetylation of AuA promotes cell invasion. Cells were seeded in inserted transwell. After 24 h, transwell was stained with crystal violet and the invasive cells were counted under microscope. *p < 0.05. Error bar indicates S.E.M (*n* = 3). Scale bar, 50 μm. (**C**) Acetylation of AuA is upregulated in migrating cell. Cells were scratched to activate cell migration and the lysates were immunoprecipitated with anti-Lys-Ac antibody or p-Ser/Thr and immunoblotting using anti-AuA antibody. (**D**) Acetylation of AuA activates AKT by p38 signaling but not ERK or FAK. Expression level of target proteins were analyzed by western blot and quantified by Image J program. Vinculin was used as loading control. *p < 0.05; **p < 0.005. Error bar indicates S.E.M (*n* = 3).

Besides its mitotic functions, AuA was reported to exert non-mitotic effects on cell movement and neurite elongation [36, 37]. The observation that AuA and ARD1 colocalized in migrating cells prompted us to explore the role of AuA acetylation in cell movement. A wound healing assay was implemented, in which a scratch was created in a monolayer of cells, and the closure rate was measured. Defect in AuA acetylation decelerated the wound closure remarkably, demonstrating that AuA acetylation is essential for cell migration (Figure 6A). Furthermore, a transwell invasion assay, wherein cells were seeded on matrigel and stimulated to invade into the lower chamber through the matrigel, was performed. The number of WT AuA-overexpressing cells that invaded the lower chamber was notably higher than that of mutant AuA-expressing cells (Figure 6B), suggesting that AuA acetylation regulated cell movement. Indeed, along with an increase in phosphorylated AuA, acetylated AuA levels were elevated significantly (Figure 6C). To clarify the molecular mechanism of cell migration regulation by K75/K125 acetylation, we analyzed AKT protein expression levels. Although AKT levels remained unchanged in both WT AuA- and K75R/K125R mutant AuA-overexpressing cells, phosphorylated AKT was augmented in WT AuA-overexpressing cells. AKT can be activated by several signaling pathways. We then tested expression level of p-38, p-ERK and p-FAK which are activators of AKT. In our study, AuA acetylation induced the phosphorylation of AKT via p38, but not via FAK or ERK signaling as phosphorylation level of p38 was elevated in WT AuA overexpressing cells, while the augmented phosphorylation of p38 was not observed in the mutant AuA overexpressing cells (Figure 6D). Because MMP-2 is downstream of AKT and crucial for cell migration and cell invasion, we measured MMP-2 expression levels. We noticed that MMP-2 expression was upregulated when cells exhibited ARD1-mediated AuA acetylation (Figure 6D), suggesting that K75/K125 acetylation regulates cell migration via the p38/AKT/MMP-2 axis.

## DISCUSSION

**Figure 7:**
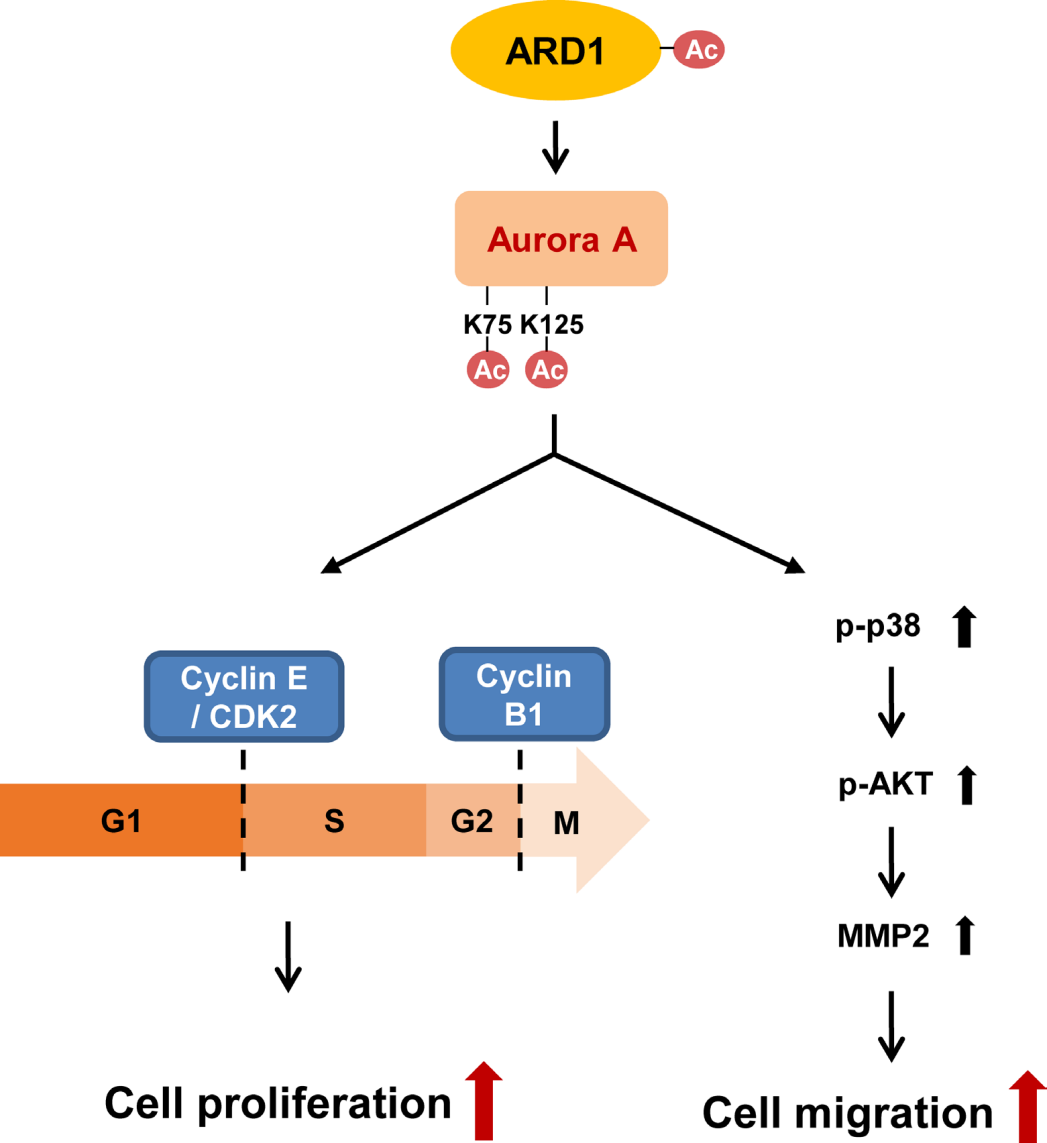
Schematics for regulation of cell proliferation and migration by ARD1-mediated AuA acetylation AuA is acetylated by ARD1 at lysine 75 and 125. Then, acetylation of AuA activates cyclin E/CDK2, promoting G1/S transition on one hand. On the other hand, acetylation of AuA increases cyclin B1 level, promoting G2/M transition, therefore, enhancing cell proliferation. In addition, acetylation of AuA by ARD1 stimulates p38 signaling, activating AKT-induced MMP-2, thus, promotes cell migration.

To the best of our knowledge, this study is the first to identify post-translational modification of AuA by the acetyltransferase ARD1. We propose a mechanism by which AuA acetylation regulates cell proliferation and migration. AuA is acetylated on lysine residues at positions 75 and 125, resulting in the activation of its kinase activity. This leads to stimulation of cell cycle progression by cyclin E/CDK2 and cyclin B1, promoting cell proliferation on one hand and activation of p38/AKT/MMP-2 pathway on the other hand, thereby stimulating cell migration (Figure 7).

AuA phosphorylation has been well studied for its role in activating AuA kinase activity, thereby inducing signaling cascades in mitotic as well as non-mitotic events [11, 37–39]. In addition to phosphorylation, a novel mechanism for the modulation of AuA function via acetylation by ARD1 was identified in this study (Figures 2B, 5D, 6D). Here, AuA was shown to colocalize with ARD1 at the centrosome (Figure 1A), and the acetylation of AuA at K75 and K125 located at N-terminus was shown to be modulated by ARD1 (Figure 3). The structure of the N-terminal domain of AuA has not been fully characterized yet, as it is difficult to crystalize the full-length AuA protein [40]. Moreover, the role of the N-terminal domain in AuA function is not clearly identified. It is reported that the N-terminus of AuA regulates its binding to partner proteins and orients AuA to the mitotic spindle. The members of the Aurora kinase family differ in their N-terminal domains, although sharing 70% homology in their C-terminal domains. The acetylation sites of AuA in the N-terminal domain probably contribute to the regulation of AuA and its localization during mitosis, and distinguish the roles of AuA in mitosis from those of other Aurora kinase family members. Whether AuA N-terminal domain assists the C-terminal domain in catalyzing the phosphorylation of its substrates is still controversial [9, 41]. Nonetheless, the kinase activity of AuA is dependent upon the binding partner and modification sites. In our study, ARD1 was shown to interact with AuA and to acetylate AuA at K75 and K125. Acetylation of K125, which is located near the ATP-binding region of AuA, probably caused conformational changes in the protein, leading to the positive regulation of AuA catalytic activity (Figure 4). It is likely that acetylation of AuA by ARD1 could promote self-phosphorylation of AuA.

Cell proliferation is important for development as it enables organs to attain the appropriate size, and exhibit normal morphology and function. As cells differentiate, their proliferation rate decreases. Uncontrollable cell proliferation results in tumorigenesis and cancer development. The balance between cell division and cell loss maintains proper cell proliferation. Therefore, the sophisticated regulatory mechanism of cell division plays a decisive role in cell proliferation and tumor growth. Cell cycle checkpoints are strictly controlled by multiple complicated mechanisms. Cyclin E/CDK2 is a key regulator of G1/S transition. Upon activation, cyclin E/CDK2 phosphorylates and inactivates retinoblastoma protein, which induced the release of E2F resulting in G1/S transition. The overabundance of cyclin E/CDK2 activity boosts G1/S transition [42]. Cyclin B1 is another switch in cell cycle progression, and the excessive expression of cyclin B1 triggers many subcellular events, resulting in G2/M transition [43]. In this study, we demonstrated that acetylation of AuA at K75 and K125 activates the expression of cyclin E, CDK2, and cyclin B1 (Figure 5D). Thus, activation of cyclin E/CDK2 and cyclin B1 by ARD1-mediated AuA acetylation promotes cell proliferation. Additionally, p53 tumor suppressor involved in multiple cell cycle checkpoints was downregulated (Figure 5D). p53 expression arrests cells at the G1/S and G2/M transitions. In the S phase, p53 expression ensures that cells do not enter mitosis in absence of DNA replication [44]. Hence, the AuA-induced p53 reduction drives cell division, promoting cell proliferation.

The AKT pathway is a key signaling pathway in the regulation of physiological processes [45, 46]. Overexpression of AKT has been described in many types of cancer cells [47–49]. AKT is activated by phosphorylation, and it is able to phosphorylate and activate downstream substrates, stimulating cell migration [50]. Besides the conventional function of AuA in the mitotic regulation of the cell cycle, the non-mitotic roles of AuA in cell migration and tumorigenesis have attracted considerable attention recently [16, 36, 51, 52]. Consistent with previous reports, our study demonstrated that ARD1-mediated AuA acetylation induces the expression of phosphorylated AKT to activate cell migration, and it provides a new regulatory mechanism by which AuA facilitates cell movement. Furthermore, MMP-2, a member of the matrix metalloproteinase family, contributes to cancer owing to its importantrole in degrading structural components of the extracellular matrix, thus paving ways for cells to migrate and invade to further locations [53]. In this study, we showed that the acetylation of K75/K125 of AuA is required for stimulating AKT signaling, resulting in MMP-2 induction to promote cell movement (Figure 6).

In conclusion, our study shows that acetylation is a novel modification of AuA and that AuA acetylation stimulates cell proliferation and cell movement. Our findings suggest a possibility that AuA acetylation is a promising therapeutic target for anticancer drug development.

## MATERIALS AND METHODS

### Antibodies

Anti-AuA antibody (ab13824) and Anti-phospho Serine/Threonine (ab17464) antibody were purchased from Abcam. Anti-acetylated lysine (Lys-Ac) antibody (#9941), anti-AKT antibody (#4691S), anti-phospho AKT (S473) antibody (#9271), anti-p38 MAPK antibody (#9212), anti-phospho p38 MAPK antibody (#4511), anti-p44/42 MAPK (Erk1/2) antibody (#9102) were from Cell Signaling Technology. Anti-cyclin B1 antibody (GNS1, sc-245), anti-cyclin E antibody (HE12, sc-247), anti-CDK2 antibody (9E10, sc-40), anti-Vinculin antibody (H300, sc-5573), anti-GFP antibody (B2, sc-9996), anti-MMP2 antibody (H-76, sc-10736), anti-GAPDH antibody (G-9, sc-365062), anti p-ERK antibody (E-4, sc-7383), anti-CDK2 antibody (D-12, sc-6248) were purchased from Santa Cruz. Anti-RFP tag antibody (MA5-15257), anti-phosphor-FAK (S397) antibody (44-624G), and anti-FAK antibody (610087) were obtained from ThermoFisher Scientific, Invitrogen and BD Biolegend respectively.

### Plasmid construction

Oligonucleotide primers were designed to amplified ARD1 gene (GenBank: NM_003491.3) and AuA gene (GenBank: NM_198433.2). Each primer covers additional sequences of restriction enzyme. ARD1 PCR product was digested and cloned into pET28b, pEGFP-C3 and pGEX-4T3. AuA PCR products were digested and cloned into pTagRFP-C and pET28b. Deletion mutants of His-AuA were constructed from pET28b-AuA plasmid. cDNAs of AuA corresponding to 1–140 aa, and 126–403 aa were amplified by PCR and inserted into pET28b.

### Cell culture

Human embryonic kidney HEK293T cell line and human breast cancer MCF7 cell line obtained from ATCC were grown in DMEM medium containing 10% FBS (fetal bovine serum) with penicillin and streptomycin in a 5% CO_2_ humidified atmosphere at 37°C. Cells were transfected using Polyethylenimine (PEI) reagent following manufacturer’s recommendation. For MCF7 stably overexpressing GFP-ARD1, RFP-AuA, MCF7 cells were transfected with pEFGP-C3-ARD1 and pTagRFP-C-AuA using Lipofectamine 2000 (Lifetechnologies) then selected by using G418 (geneticin).

### Cell synchronization

MCF7 cells were synchronized by treated with cytosine arabinoside in 16 h for G1/S phase. After cells were released into fresh medium in 4 h, cells were then arrested in early mitosis by using nocodazole at concentration 100 ng/ml for 6 h. For metaphase, cells were then treated with MG132 for 30 min. And cells were in anaphase and telophase after being released from MG132 30 min and 60 min respectively.

### Immunoflourescence

Cells were grown on circular glass coverslips plated in 24-well plate. Cells were fixed with formaldehyde in 10 min, permeabilized by incubating in PBS containing 0.25% Triton-X in 10 min and washed 3 times by PBS, then blocked for 1 h in 1% BSA. Primary antibody recognizing AuA (1:500) was diluted in blocking buffer and incubated overnight at 4^°^C. Alexa Flour^®^546 goat anti-mouse antibody was used at recommended concentration and incubated in 1h in the dark at room temperature. This was followed by counterstaining with Hoesch and mounting with Gel/mount (Biomeda Corp.). Cells were observed under Axiovert M200 microscope (Zeiss).

### Immunoblotting and immunoprecipitation

Total cell lysates were isolated using cell lysis buffer (Cell signaling), which contained 20 mM Tris-HCl pH7.5, 150 mM NaCl, 1 mM Na2EDTA, 1 mM EGTA, 1% Triton, 2.5 mM sodium pyrophosphate, 1 mM beta-glycerophosphate, 1 mM Na3VO4, 1 μg/mi leupetin and the protease inhibitor cocktail set (Calbiochem). Lysates were separated by sodium dodecyl sulfate gel electrophoresis, and transferred to nitrocellulose membranes for Western blot analysis. Detection was performed using ECL (enhanced electrochemiluminescence) plus (Amersham Bioscience). For immunoprecipitation, lysates were immunoprecipitated with appropriate primary antibody. The immunoprecipitated complexes were subjected to electrophoresis and immunoblotting. Each experiment was repeated at least 3 times.

### *In vitro* acetylation assay

BL21 cells transformed with plasmids pET28a-ARD1, pGEX-4T3 or pET28a-AuA were grown to and OD600 of 0.6–0.8. Overall, 1 mM IPTG was added to induce His-ARD1, GST-ARD1 or His-AuA, then cells were grown for overnight at 25^°^C. For His-tagged protein purification, cells were collected and proteins were extracted with a lysis buffer (pH 7) containing 50 mM NaH2PO4, 300 mM NaCl, 10 mM Imidazole and 1% Triton X-100. His-tagged proteins were purified with Ni-NTA and followed by eluted with an elution buffer containing 50 mM NaH2PO4, 300 mM NaCl, and 250 mM Imidazole. For GST-tagged protein purification, cells were collected and proteins were extracted with lysis buffer containing 250 mM Tris-Cl pH 8.5, 500 mM NaCl, 5 mM EDTA and 1% Triton X-100. Cell lysates were then incubated with Glutathione-Agarose for 4 h, following by washing three times with PBS to obtain purified GST-tagged protein. Acetylation assay was performed as described. Freshly prepared recombinant ARD1 and AuA were incubated in the reaction mixture [50 mmol/L Tris-HCl (pH 8.0), 0.1 mmol/LEDTA, 1mmol/LDTT, 10% glycerol, and 10 mmol/L acetyl-CoA] at 37^°^C. Each experiment was repeated at least 3 times.

### ATP-binding assay

Total cell lysates were isolated using cell lysis buffer (Cell signaling). 1mg cell lysates were then subjected to ATP-agarose resins (Innova Biosciences) and incubated at 4^°^C for 4 h. The ATP-bound protein complexes were washed three times with the same buffer. Proteins bound to ATP were eluted by sample buffer and subjected to electrophoresis and immunoblotting. Each experiment was repeated 3 times.

### ATPase activity assay

Recombinant proteins were applied to *in vitro* acetylation assay and ATPase activity were measured using High Throughput Colorimetric ATPase Assay Kit (Innova Biosciences) according to the manufacturer’s instructions. Briefly, recombinant proteins were incubated for 30 min at 30°C in a reaction buffer consisting of 50 mM Tris (pH 7.5), 2.5 mM MgCl2 and 0.5 mM ATP. After that PiColorLock Gold reagent and Accelerator were added to the mixture. Then Stabilizer was added and stabilized for 30 min. The absorbance at 595 nm was measured. Each experiment was repeated 3 times.

### Protein sequence alignment

Protein sequence alignment was conducted using Clustal Omega.

### Cell proliferation assay

Cells were seeded in 96-well plate at a density of 10^3^ cells per well and cultured for 48 h. The proliferation rates were measured using a Non-Radioactive Cell Proliferation Assay Kit (Promega) according to the manufacturer’s instructions. Briefly, 20 ul of freshly mixed tetrazolium/phenazine methosulfate was added, and the cells were incubated for 1 hour to allow color development. The absorbance at 490 nm was measured to indicate the number of viable cells. Each experiment was repeated 3 times.

### Clonogenic assay

Cells were seeded in 6-well plate at a density of 10^3^ cells per well and cultured for 7 days to form colonies. Colonies then were fixed with 1% formaldehyde and stained with crystal violet (0.5%) and counted. Each experiment was repeated 3 times.

### Cell cycle analysis

2 × 10^6^ cells were harvested and washed twice with PBS, fixed overnight with 70% ethanol. After two washes with cold PBS, cells were incubated with RNAse for 30 min, following up with 30 min incubation with propidium iodine. Cell cycle was analyzed by BD Calibur flowcytometry. Each experiment was repeated 3 times.

### Wound healing assay

Wounds were created in confluent MCF7 cells by 200 μl tip and washed twice by PBS to remove non-adherent cells. To inhibit the influence of cell proliferation, cells were treated with 10 μM cytosine arabinoside. After 24 h incubation, the wound closure rates were measure under a phase-contrast microscope. Each experiment was repeated 3 times.

### Transwell invasion assay

Invasion assays were conducted using transwell invasion chambers coated with Matrigel matrix (Corning). Cells were seeded in the upper chambers (10^4^ cells/well) in FBS-free DMEM. DMEM containing 10% FBS was added to the lower chambers. After 24 h of incubation, invaded cells on the lower surface were stained with crystal violet stain and counted under a light microscope. Each experiment was repeated 3 times.

### Statistical analysis

Data analysis and statistical significance were tested using Graphpad Prism 6.0. *P* values were calculated by applying two-tailed Student’s *t* test. Statistical significance was determined at *P* < 0.05.

## SUPPLEMENTARY MATERIALS FIGURE



## Abbreviations

AuA: Aurora kinase A
ARD1: Arrest defective protein 1
D-box: destruction box
DAD: D-box activating domain
