# Supplementary material for: ARD1-mediated aurora kinase A acetylation promotes cell proliferation and migration
Source: Oncotarget. 2017 Jul 18;8(34):57216–30. doi: 10.18632/oncotarget.19332 (PMC5593637; doi:10.18632/oncotarget.19332)
Supplement: Supplementary file 1 [file oncotarget-08-57216-s001.pdf]

ARD1-mediated aurora kinase A acetylation promotes cell proliferation and migration

SUPPLEMENTARY MATERIALS

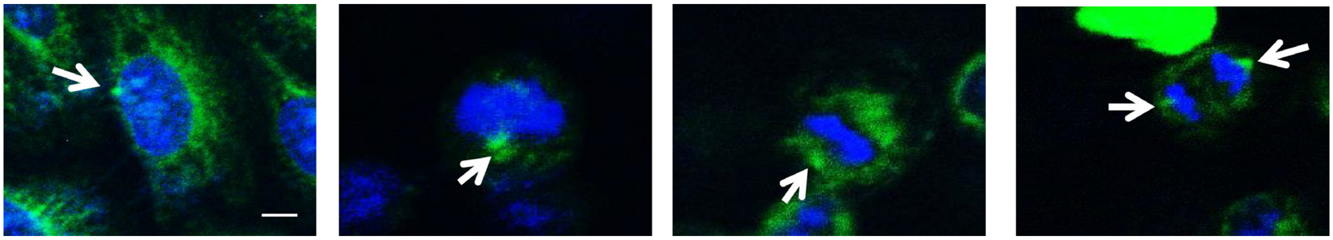

Supplementary Figure 1: Centrosome-like localization of ARD1. Overexpressing GFP-ARD1 cells were synchronized, fixed and then mounted. Cells were then visualized by confocal microscope. DNA was counter-stained by Hoechst. Scale bar, 5 μm. Subcellular localization of ARD1 is indicated by arrows.
